# Antioxidant and antibacterial properties of coating with chitosan–citrus essential oil and effect on the quality of Pacific mackerel during chilled storage

**DOI:** 10.1002/fsn3.958

**Published:** 2019-02-11

**Authors:** Yuan Li, Chunhua Wu, Tiantian Wu, Chunhong Yuan, Yaqin Hu

**Affiliations:** ^1^ National Engineering Laboratory of Intelligent Food Technology and Equipment Key Laboratory for Agro‐Products Postharvest Handling of Ministry of Agriculture Key Laboratory for Agro‐Products Nutritional Evaluation of Ministry of Agriculture Zhejiang Key Laboratory for Agro‐Food Processing Fuli Institute of Food Science College of Biosystems Engineering and Food Science Zhejiang University Hangzhou China; ^2^ Marine Research Center of Zhoushan Zhejiang University Zhoushan China; ^3^ College of Food Science Fujian Agriculture and Forestry University Fuzhou China; ^4^ Department of Food Production and Environmental Management Faculty of Agriculture Iwate University Morioka Japan

**Keywords:** chitosan, citrus essential oil, Pacific mackerel, quality

## Abstract

The goal of the study was to investigate whether chitosan–citrus essential oil composite works as an efficient preservative in Pacific mackerel (*Pneumatophorus japonicus*) during chilling storage. FT‐IR analysis showed that chitosan–citrus essential oil coating was successfully prepared. Our results demonstrated that chitosan–citrus essential oil coating possessed significantly higher capability of scavenging reactive oxygen species (${\text{O}}_{2}^{-}$ and OH−) than chitosan. Furthermore, Pacific mackerel coated with chitosan–citrus essential oil composite could significantly reduce parameters of corruption including physicochemical (drop loss, biogenic amine, and thiobarbituric acid‐reactive substances) and microbiological parameters (total viable count), as compared with untreated and chitosan groups after 12 days of storage at −3°C. These results indicated that CS‐CEOs could work as efficient preservative for Pacific mackerel storage through ameliorating redox state and inhibiting microbial growth and suggested that chitosan–citrus essential oil composite has great potential in preservation of aquatic products during superchilled storage.

## 1 INTRODUCTION

Pacific mackerel (*Pneumatophorus japonicus*) is an abundant species of pelagic fish and spoils easily. Thus, how to maintain its freshness is a predominant issue for researchers (Cao et al., 2013). Up to now, the methods to keep the freshness of aquatic products have been well‐developed, including low temperature, biochemical preservation, high pressure, the modified atmosphere, irradiation, and ozone preservation (Wu et al., 2014). Low‐temperature storage is the most common method used in the preservation of fresh aquatic products, but deteriorative changes still occur during the process of freezing, storage, and thawing, leading to the changes in flavor, odor, texture, and color (Tironi, Tomás, & Añón, 2010). Bacteria keep growing during freezing storage, either (Carlez, Rosec, Richard, & Cheftel, 1994; Dainty & Mackey, 1992). Superchilled storage combined with biopolymers is one of the ideas currently applied. As polymer coating materials have the advantage of preserving water as well as the properties of antioxidant and antibacterial, there have been a broad range of applications in the field of food preservation. Among these polymer coating materials, chitosan (CS) is a biopolymer with a wide range of bio‐applications.

Chitosan is a deacetylated derivative of chitin, and it is a kind of natural polysaccharide with biological macromolecules in which α‐amino‐d‐glucose connect to β‐1,4‐glycosides and bond. Study of Jeon, Park, and Kim (2001) showed that 0.1% of chitosan (degree of deacetylation: 89%, average molecular weight: 685,000) definitely inhibited gram‐positive bacteria including *Streptococcus mutans*,* Micrococcus luteus*,* Staphylococcus aureus*,* Staphylococcus epidermidis*, and *Bacillus subtilis*, and the minimum inhibitory concentrations for lactic acid bacteria were <0.03%, while 0.1% of chitosans with the molecular weight of 28–1,671 kDa were proved to have the minimum inhibitory concentrations of 0.08% for *Bacillus megaterium* and *Bacillus cereus* (No, Park, Lee, & Meyers, 2002). Tsai, Su, Chen, and Pan (2002) observed that the minimal lethal concentrations of chitosan ranged between 50 and 200 ppm for *B. cereus*,* Escherichia coli*,* Listeria monocytogenes*,* Pseudomonas aeruginosa*,* Shigella dysenteriae*,* S. aureus*,* Vibrio cholerae*, and *V. parahaemolyticus* and 200 ppm and 500 ppm for *Candida albicans* and *Fusarium oxysporum*, respectively.

There have been many researches focusing on the use of chitosan or essential oil to extend the shelf life of aquatic products during storage. Fernández‐Saiz, Sánchez, Soler, Lagaron, and Ocio (2013) observed that chitosan films had an inhibition effect of refrigerated sole and hake fillets packaged in air and under vacuum. Bingöl, Bostan, Uran, Alakavuk, and Sivri (2015) indicated that the moisture loss could be reduced for *Parapenaeus longirostris* coated with chitosan. Souza et al. (2010) observed that chitosan‐based coating could extend the shelf life of salmon for 3 days. Four plant essential oils including clove leaf oil, clove bud oil, rosemary oil, and thyme oil incorporated alginate gels were proved to better control the quality of *Pangasianodon hypophthalmus* fillets by Rao, Jesmi, and Viji (2017). Matan (2012) proved that essential oils including cinnamon oil, clove oil, anise oil, turmeric oil, guava leaf oil, nutmeg oil, and lime oil incorporated with edible film could extend the shelf life of dried fish (*Decapterus maruadsi*).

Some studies have also been researched on the combination of chitosan‐essential oil in films in extending shelf life of fish products. Remya et al. (2016) observed that chitosan incorporated with ginger (*Zingiber officinale*) essential oil could efficiently keep the quality of chilled stored barracuda (*Sphyraena jello*) fish. Edible coatings of chitosan in combination with carvacrol were proved to delay the emergence of total volatile bases in ice‐stored tilapia (*Oreochromis niloticus*) fillets. Chamanara, Shabanpour, Khomeiri, and Gorgin (2013) showed that chitosan coating with thyme essential oil was able to prolong the shelf life of rainbow trout (*Oncorhynchus mykiss*) for approximately 6 days. Gómez‐Estaca, De Lacey, López‐Caballero, Gómez‐Guillén, and Montero (2010) observed complex gelatin–chitosan film incorporating clove essential oil could drastically reduce gram‐negative bacteria in fish fillets during chilled storage.

Citrus essential oil is a kind of essential oil extracted from the peel of citrus, with excellent antibacterial and antioxidant properties (Djenane, 2015; Javed et al., 2014). It is reported that citrus essential oil (1:2 diluted by ethanol) can inhibit the growth of yeast, fungi, spore bacteria, and food toxin‐producing bacteria (Deans & Ritchie, 1987). Zohra et al. (2015) studied the chemical composition of the essential oils of four Algerian citruses, indicating that essential oils could be used as natural fungicides against phytopathogenic fungi. Randazzo et al. (2016) evaluated the antimicrobial activity of eight essential oils extracted from the fruit peel of citrus genotypes against 76 strains of *L. monocytogenes* and concluded that lemon essential oils incorporated into chitosan films could be an efficient tool to control *L. monocytogenes*, especially under refrigerated conditions.

As a byproduct of citrus, a fruit widely distributed in temperate zones, citrus essential oil is easy to obtain, and the cost of extraction is relatively low, and it is proved to be an economical natural oxidant. However, citrus essential oil is easy to volatilize and oxidize when exposed to air; thus, combining essential oil with chitosan could be an economical and easy‐realizable way to prolong the shelf life of aquatic products. Thus, this study aims to evaluate the antioxidant and antibacterial ability of chitosan–citrus composite coating, and investigate its effect on the preservation of Pacific mackerel. Briefly, chitosan was used as the matrix material to form a thin film on the surface of Pacific mackerel. According to Duun and Rustad (2007), most foodstuffs hold the initial freezing point at −0.5~−2.8°C, and 1–2°C below the freezing point, the fish products could be partial freezing, and its shelf life could be longer than the traditional 4°C storage. In order to observe the influence of chitosan–citrus essential oil over a longer period, all samples are stored at −3°C. Lipid oxidation and protein denaturation to Pacific mackerel during superchilled storage were studied. The results would contribute to the research for an effective and low‐cost preservation method of Pacific mackerel.

## 2 MATERIALS AND METHODS

### 2.1 Materials

Pacific mackerel (28–30 cm in size, weight 230–250 g) were purchased at local fishery market (Zhoushan, China) and transported back to the laboratory in ice. Chitosan with deacetylation degree of 95% and molecular weight of 80–90 KDa was purchased from Qingdao Yunzhou Biotechnology Co. Ltd, China. Citrus essential oil was purchased from Flowers Shop TM (Shanghai, China) and encapsulated in a glass bottle under 4°C. Analytical grade chemical reagents were purchased from Sinopharm Group Chemical Reagent Co., Ltd., (Shanghai, China).

### 2.2 Design of experiments

The chitosan–citrus essential oil coating solution was prepared using the following method. According to previous research (Giatrakou, Ntzimani, & Savvaidis, 2010; Ruan & Xue, 2002; Wu, Fu, et al., 2016; Wu, Li, et al., 2016; Wu, Wang, et al., 2016), the 1.5% (w/v) chitosan was used as materials to prepare chitosan solution in 1% (v/v) acetic acid. Citrus essential oil was mixed with the chitosan solution to obtain a final citrus essential oil concentration of 1.5% (w/v), adding 0.2% (v/v) Tween 80 as the emulsifier. The mixture was stirred for 16 hr at 4°C before use.

Pacific mackerel samples were divided into three treatment groups: (1) control group without any coating treatment (CK), (2) samples coated with chitosan (T1), and (3) samples coated with chitosan–citrus essential oil composite (T2).

Pacific mackerel of similar size and shape were randomly divided into three groups (CK, T1, and T2), and after gutted and cleaned, each group has 15 fish samples. T1 and T2 were soaked in ice‐bathed chitosan solutions and in the chitosan–citrus essential oil solutions for 20 min, respectively. CK was soaked in water for the same time for comparison. The samples were then preserved in refrigerator under −3°C.

Each group of Pacific mackerel was selected randomly. Triplicate samples were taken out to perform the physical and chemical analyses on day 0, 3, 6, 9, 12, and 15.

### 2.3 Chemical composition of citrus essential oil (CEOs)

GC‐MS (7890/5975, Agilent Technologies, Palo Alto, USA) was carried out to analyze the chemical composite of CEOs. The electron impact ionization mode mass spectrometer operated in was at a voltage of 70 eV. The mass scan range was 40–400 amu. The flow rate of the helium carrier gas on HP‐5 column (30 m × 0.25 mm) was 1 ml/min. The chromatographic column is a fused silica capillary column HP‐5 (30 m × 0.25 mm). The analysis performed in the splitless mode, and injector temperature was 250°C. The column was held at 40°C for 1 min, and then increased from 40 to 220°C at 3°C/min, held at 220°C for 25 min, and finally increased to 280°C at a rate of 20°C/min, then held for 3 min. The identification of the individual compounds was based on the comparison of their relative retention times with those of authentic samples on the capillary column and by matching their mass spectra of peaks with available Wiley and NIST. The relative content of each component was calculated by area normalization method.

### 2.4 Fourier transform infrared spectrophotometry (FT‐IR) of films

FT‐IR spectra were recorded using a Thermo Fisher Nicolet. KBr disks of samples were prepared, and the analyzed wavelength range was 4,000–400 cm^−1^.

### 2.5 Determination of antimicrobial activity

Escherichia coli O157 (*E. coli*) and Listeria monocytogenes CICC21633 (*L. monocytogenes*) were used as the test bacteria. All samples were dissolved in 1% acetic acid. The antimicrobial activity was evaluated by the inhibition zone assay in agar medium with Oxford cup method, and 200 μl of sample was loaded on each oxford cup (Hu et al., 2017).

### 2.6 Determination of superoxide anion radical scavenging activity

Take 4.5 ml Tris‐HCl (pH 8.2), which had been preheated under 25°C for 25 min, add chitosan–citrus solution of 1 ml, and 25 mmol/L pyrogallol solution of 0.4 ml, after mixing the reaction at 25°C for 5 min, terminate the reaction by adding 1 ml hydrochloric acid solution of 8 mol/L, and the absorbance was measured at 299 nm wavelength. The control group and the blank group were added with the same volume (1 ml) ethanol and ascorbic acid solution instead of the sample, respectively. Samples of superoxide anion radical scavenging (E) were calculated by formula (1).

(1) $$\text{E}/\%=\left({\text{A}}_{0}-{\text{A}}_{i}\right)/{\text{A}}_{0}\times 100$$

In formula (1), A_0_ represented the absorbance of the control group; A_i_ represented the absorbance of the sample.

### 2.7 Determination of hydroxyl radical scavenging activity

The hydroxyl radical scavenging activity of materials was measured using the method of Liu, Chen, Kong, Han, and He (2014). The reactive sulphydryl content (R) was calculated using formula (2).

(2) $$\text{R}\;\left(\text{mmol}/\text{kg}\right)=73.53\times \left({\text{A}}_{412}-1.6934\times {\text{A}}_{532}+0:009932\right)$$

In formula (2), A_412_ and A_532_ represented the absorbance of the assay, where A_412_ and A_532_ are the absorbance of the assay solution at 412 and 532 nm, respectively.

### 2.8 Determination of drop loss, pH, total volatile basic nitrogen, biogenic amine, and thiobarbituric acid‐reactive substances

A 3.0 g sample was parceled in filter paper (filter hole 0.3–0.5 μm) and placed in a dry tray for 2 hr. The drop loss can be calculated by comparing the initial and the final mass of sample. The pH values of the samples were measured using a Testo 205 pH meter (Testo AG, Lenzkirch, Germany) by inserting an electrode into the dorsal muscle. The total volatile basic nitrogen (TVB‐N) was determined according to the method of Chomnawang, Nantachai, Yongsawatdigul, Thawornchinsombut, and Tungkawachara (2007). Biogenic amine (BA) was measured according to the method of Wu, Fu, et al. (2016), Wu, Li, et al. (2016) and Wu, Wang, et al. (2016). The thiobarbituric acid‐reactive substances (TBARS) contents were determined according to the method of Kim, Yim, and Choi (1995).

### 2.9 Determination of the total viable count (TVC) and total psychrotrophic count (TPC)

The measurement of TVC was modified from Wang, Wang, Liu, and Liu (2012), Rao et al. (2017) and Ojagh, Rezaei, Razavi, and Hosseini (2010). Approximately 5.0 g of Pacific mackerel sample was homogenized with 45 ml of sterile 0.9% (w/v) normal saline (NS). The resulting suspensions were serially diluted (1:10) in sterile NS for bacteriological analysis. The total microbial counts were determined by the pour plate method using plate count agar. For TVC, the inoculated plates were incubated at 37°C for 48 hr. For TPC, the inoculated plates were incubated at 7°C for 10 days. Results were showed by the logarithmic of total number of colonies (lg cfu/g).

### 2.10 Sensory evaluation

The appearance, odor, and organization of Pacific mackerel are evaluated by 37 trained food professionals. The evaluation criterion is shown in Table 1.

**Table 1:** Sensory evaluation standards of pacific mackerel

Items	Evaluation standard			
	Extremely love 9	Like 6–8	Dislike 2–5	Extremely dislike 1–2
Appearance	Of normal color, the section of the muscles is glossy, the color is evenly distributed, and the surface is bright, not with too much water and smooth.	Of normal color, the section of the muscles is glossy, and the surface is comparatively bright with a little water.	The color is slightly dim, and the section of the muscle is slightly shiny. Apparent moisture exudation on the surface.	The color is dull, dull muscle section. Extremely water loss occurs.
Odor	Strong inherent scent of pacific mackerel and of no peculiar smell.	Comparatively strong inherent scent of pacific mackerel and basically of no peculiar smell.	Slight inherent scent of pacific mackerel and of some peculiar smell.	Inherent scent of pacific mackerel disappeared with the odor of ammonia.
Organization	Muscle is solid and elastic, and sags disappear immediately after the finger press.	Muscle is comparatively solid and elastic, and sags disappear soon after the finger press.	Muscle is slightly soft, and sags disappear slowly after the finger press.	Muscle is extremely soft, and sags basically not disappear after the finger press.

*Note*

1—Extremely dislike, 2—Particularly unpleasant, 3—Repugnant, 4—Mild dislike, 5—Neutral, 6—Mild like, 7—Moderate like, 8—Like, 9—Extremely love. The average score of each people in the evaluation group was the result of comprehensive sensory evaluation.

### 2.11 Statistical analysis

Data were analyzed using analysis of variance (ANOVA), statistical correlation was evaluated by Pearson correlation coefficients, and a *p*‐value <0.05 was considered statistically significant. Correlation cofficient were performed by the program SPSS 16.0 (SPSS Inc., Chicago). Figures were drawn by SciDAVis software.

## 3 RESULTS AND DISCUSSION

### 3.1 Analysis of CEOs components

Constitutes of CEO identified and quantified by GC‐MS are shown in Table 2. In all of the 30 kinds of compounds that have been detected, Ɗ‐Limonene holds the highest proportion of 90.23%. Ɗ‐Limonene is one of the most common terpenes in nature and is considered to have low toxicity (Sun, 2007), and it has been listed as the “Generally Recognized as Safe” (GRAS) by FDA. Similar as other terpenes, it provides antioxidant and antibacterial properties to CEOs. The other four components have the content of more than 1% including myrcene, α‐terpinene, linalol, and α‐Pinene. α‐Pinene has obvious bactericidal action by inhibiting the biosynthesis of RNA, DNA, fungal polysaccharides, ergosterol, and Candida albicans. These components can decelerate the growth of microorganism, but their applications are restricted by their volatility. Thus, coating citrus essential oil into other matrix could be a promising way to broaden its application.

**Table 2:** Components of CEOs

No.	Retention time (min)	Compound	Molecular formula	% of the total peak area
1	9.82	α‐Pinene	C_10_H_16_	1.02
2	10.36	Camphene	C_10_H_16_	0.03
3	11.67	Phellandrene	C_10_H_16_	0.03
4	11.72	β‐Pinene	C_10_H_16_	0.12
5	12.79	Thujene	C_10_H_16_	0.77
6	12.88	Myrcene	C_10_H_16_	1.79
7	14.60	Ɗ‐limonene	C_10_H_16_	90.13
8	17.11	Octanol	C_8_H_18_O	0.06
9	17.13	Terpinolene	C_10_H_16_	0.08
10	17.21	α‐Terpinene	C_10_H_16_	2.08
11	17.85	Nonanal	C_9_H_18_O	0.01
12	18.01	Linalol	C_8_H_18_O	2.01
13	19.42	Carvoel	C_10_H_16_O	0.02
14	20.09	Citronellal	C_8_H_18_O	0.03
15	21.29	Terpenol	C_8_H_18_O	0.44
16	22.02	Ɑ‐Terpineol	C_8_H_18_O	0.07
17	22.49	Decyl aldehyde	C_10_H_20_O	0.26
18	23.23	Carvyl acetate	C_12_H_18_O_2_	0.03
19	23.67	Citronella	C_10_H_20_O	0.02
20	23.83	Nerol	C_8_H_18_O	0.05
21	23.88	Citral	C_10_H_16_O	0.04
22	24.27	D‐carvone	C_10_H_14_O	0.02
23	24.87	Geraniol	C_8_H_18_O	0.02
24	25.11	Cyclohexanol	C_6_H_10_O	0.04
25	27.38	Undecanal	C_11_H_22_O	0.01
26	29.64	Neryl acetate	C_12_H_20_O_2_	0.05
27	31.49	Tridecylic aldehyde	C_16_H_16_N_2_O_2_	0.02
28	34.19	Ɓ‐Elemene	C_15_H_24_	0.01
29	38.39	Octanoic acid	C_14_H_28_O_2_	0.02
30	56.00	Aurapten	C_19_H_22_O_3_	0.05

### 3.2 FT‐IR spectrum of films

**Figure 1:**
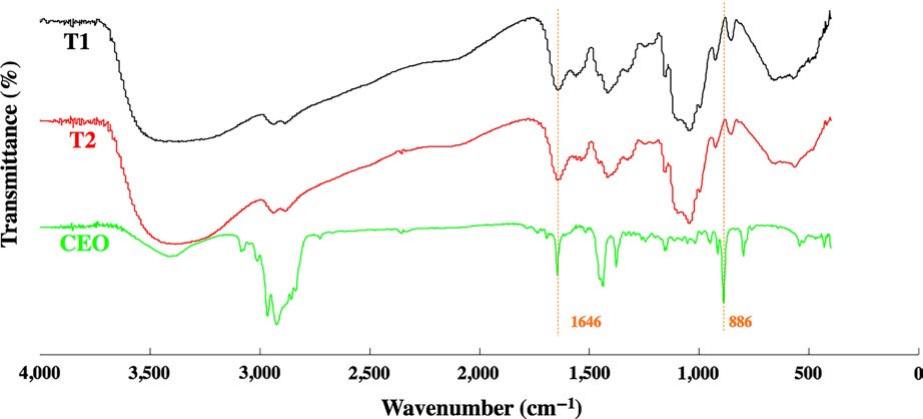
FT‐IR spectrum of CEO and films

FT‐IR, which is broadly used to identify the functional groups, was used to analyze whether CEO has been successfully mixed into CS. The FT‐IR spectrums of T1, T2, and CEO were shown in Figure 1. Both T1 and T2 showed peaks at around 2,879 cm^−1^, corresponding to N‐H stretching vibration absorption peak of chitosan. The intensity of the scissoring vibration absorption peak of O‐H at around 1,376 cm^−1^ was similar in T1 and T2. Compared with T2, the intensity of bending vibration absorption peak of N‐H at around 1,646 cm^−1^ in T1 was slightly reduced, which could be attributed to the superposition effect of CEO and CS for similar functional groups was identified in the spectrum of CEO. Conversely to this trend is that the intensity of peaks at 886 cm^−1^ decreased in T2 compared to T1, indicating the presence of CEO in T2 (Li et al., 2018). The FT‐IR spectrums showed that CS and CEO were successfully mixed in T2.

### 3.3 Superoxide anion radical scavenging activity and hydroxyl radical scavenging activity of chitosan–citrus and chitosan solution

**Figure 2:**
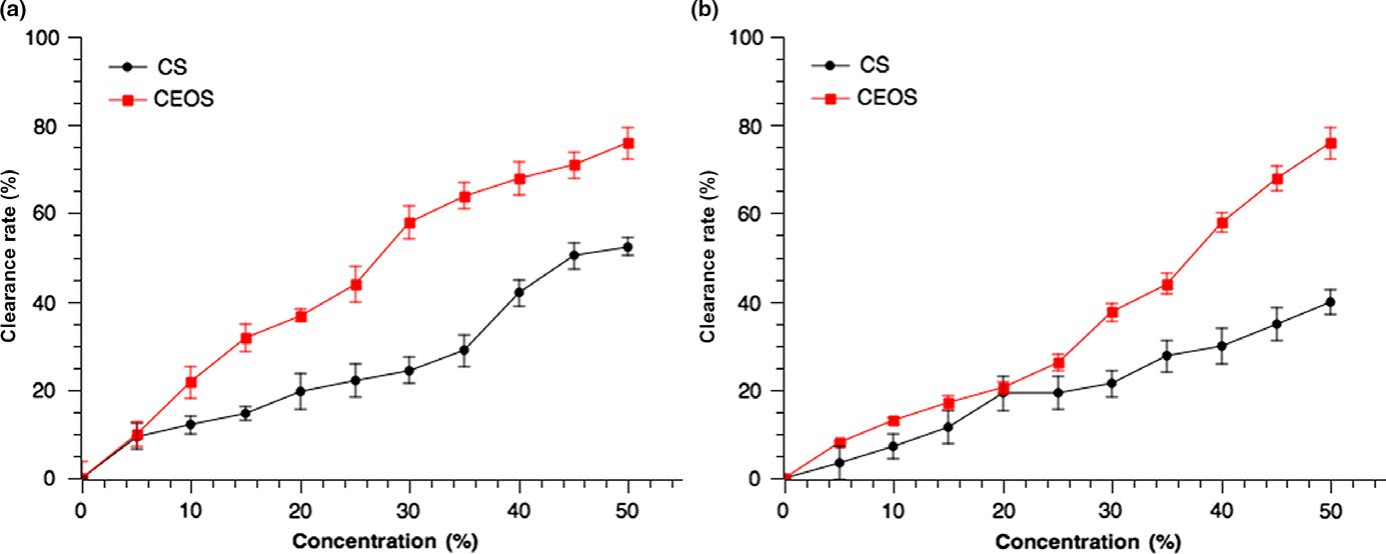
Antioxidant of chitosan–citrus composite and chitosan. In the figure, CS represents chitosan, CEOs represents chitosan–citrus composite. (a) Superoxide anion radical scavenging activity; (b) Hydroxyl radical scavenging activity

The superoxide anion is a major agent in the oxygen toxicity (Sawyer & Valentine, 1981), and it was closely related to the biological course including apolexis, inflammation, and tumor. (Sun, Xie, & Xu, 2004). Figure 2a shows the superoxide anion radical scavenging activity of both chitosan material and chitosan–citrus coating in terms of concentration. As the concentration of solution increased from 0% to 50%, the superoxide anion radical scavenging activity of both chitosan–citrus and chitosan coating agent solution increased, indicating their super oxygen‐anion free radical scavenging ability is strongly concentration dependent. This scavenging ability is substantially larger for chitosan–citrus than that for chitosan. Adding citrus to chitosan reinforces the clearance rate of superoxide anion radical. Besides, the amino groups in chitosan can react with free radicals to form most stable macroradicals, which can partly explain the scavenging effect of chitosan. Moreover, the contents of d‐Limonene in citrus essential oil are more than 90% (Wu, Fu, et al., 2016; Wu, Li, et al., 2016; Wu, Wang, et al., 2016), which contributes to the excellent performance of antioxidation as d‐Limonene is an effective hydrogen donor. The integration of citrus oil into CS enhanced the antioxidant ability in comparison with CS alone.

Among various reactive oxygen species, the chemical activity of hydroxyl radical ˙OH is the strongest (Xie, Xu, & Liu, 2001). The clearance rate of hydroxyl radical of chitosan–citrus coating and chitosan is shown in Figure 2b. The clearance rate of both chitosan–citrus coating and chitosan alone increased significantly (*p* < 0.05) with the increasing concentration of solution. The chitosan–citrus solution exhibited a significantly (*p* < 0.05) higher increase rate, reaching a clearance rate of 75.97% at the concentration of 50%. The hydroxyl radical scavenging activity of chitosan can be partly attributed to the ˙H in its structure. The more ˙H provided by chitosan, the stronger the hydroxyl radical clearance rate had. The groups in the structure of chitosan such as –NH_2_ and –OH can eliminate the hydroxyl radical by inhibiting the chain reaction of reactive oxygen radicals. Terpenoids in citrus essential oil contain double bonds, which can eliminate hydroxyl radical with ˙H by addition reaction. The enhancement of hydroxyl radical scavenging activity in chitosan–citrus solution might due to the superposition effect of chitosan and citrus in terms of hydroxyl radical scavenging.

### 3.4 Assay of the antibacterial activity

Inhibition zones of chitosan (CS) and chitosan–citrus (CS‐CEOs) against *E. coli* and *L. monocytogenes* were investigated. The inhibition zone diameters of CS against *E. coli* and *L. monocytogenes* were 12.24 ± 1.03 mm and 13.35 ± 0.79 mm, respectively, and the inhibition zone diameters of CS‐CEOs against *E. coli* and *L. monocytogenes* were 17.23 mm ± 1.29 mm and 19.19 mm ± 1.27 mm, respectively. The antimicrobial activity of CS‐CEOs was better combined with that of CS for both *E. coli* and *L. monocytogenes*. The enhancement of antibacterial property might due to the components which exert potent antimicrobial activity, such as citrullene and limonene (Di Pasqua, Hoskins, Betts, & Mauriello, 2006). Citrus essential oil exerts its bactericidal effects at the membrane level, where they increase the permeability of the cell membrane (Nannapaneni et al., 2008). It has been reported that the antibacterial property of chitosan could be due to its polycationic nature, which could interfere with the negatively charged residues of macromolecules at the surface, interacting with the membrane of the cell to alter cell permeability (Fei Liu, Lin Guan, Zhi Yang, Li, & De Yao, 2001). The antibacterial effect of chitosan–citrus essential oil composite was resulted from the synergistic effect of those two kinds of antibacterial agent.

### 3.5 The effect of chitosan–citrus coating on the preservation properties of Pacific mackerel meat during superchilled storage

The fish meat of control group completely decayed in the 12th day, so the experiment on preservation of CK stopped at this stage and the data were no longer collected for this group.

### 3.6 Drop loss (DL)

The degree of decay on dead fish can be reflected by its drop loss (DL). The increase of DL indicates the adverse changes of the fish.

**Figure 3:**
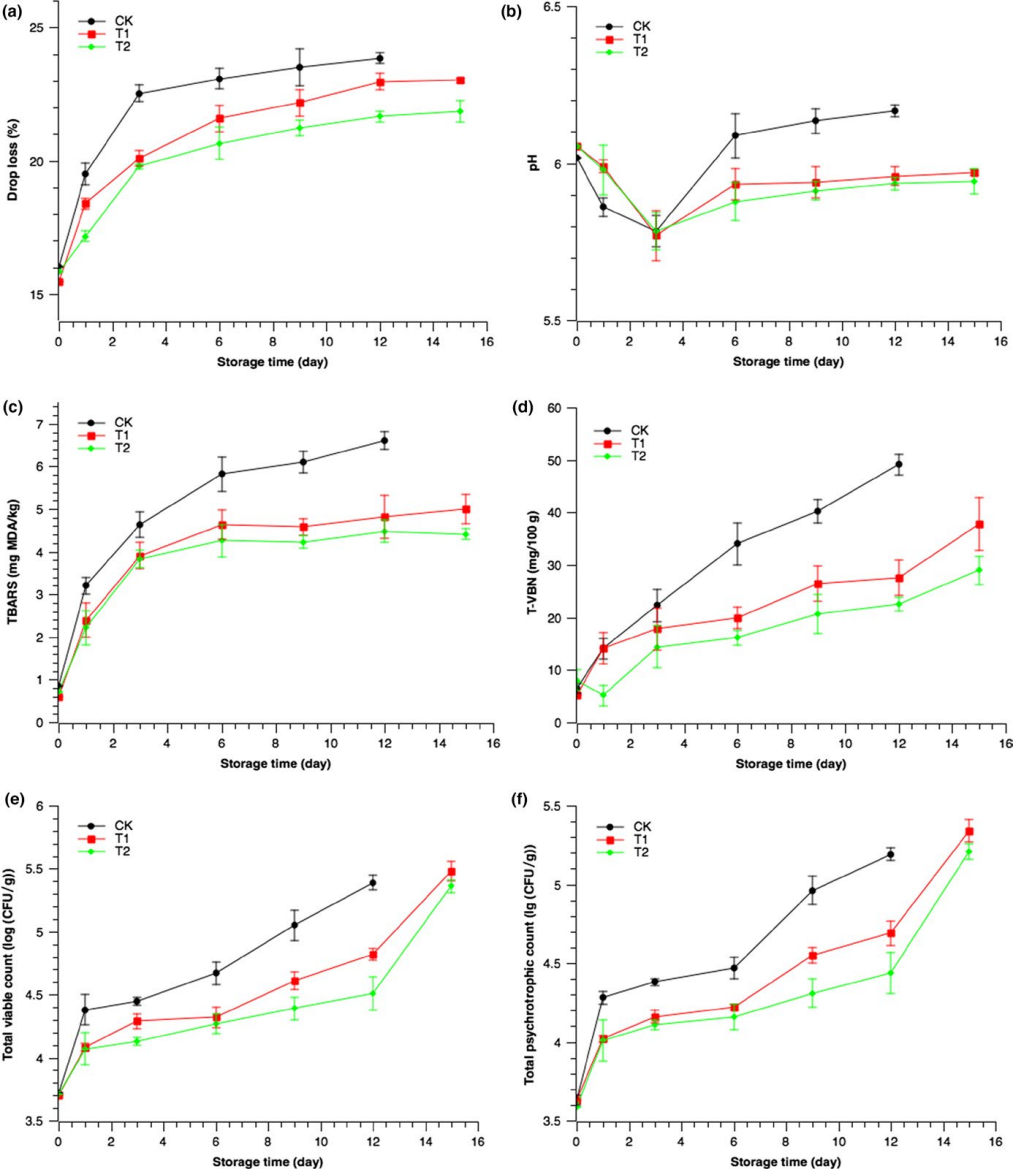
Preservation performance indices of Pacific mackerel during superchilled storage. In the figure, (a) DL; (b) pH; (c) TBARS; (d) TVB‐N; (e) TVC; (f) TPC

As shown in Figure 3a, the DL of all the three groups increased from the beginning of the superchilled storage, which indicated that the quality of Pacific mackerel gradually got worse. The fastest rising speed of DL occurred in the first 3 days of superchilled storage, and during this period, the rising speed of DL in CK was significantly faster than the other two groups (*p *<* *0.05). The rising speed of DL slowed down later, and T1 and CK showed the same trend that slightly higher than T2 (*p *<* *0.05), indicating that the chitosan has slight effects on DL of refrigerated Pacific mackerel. The chitosan–citrus composite coating helped to hold the water in Pacific mackerel. Possibly, the functional groups of citrus essential oil interact with the hydroxyl and amino groups of chitosan, causing the decrease of free space in chitosan molecules and its molecular mobility; therefore, the chitosan–citrus composite coating has a strong barrier property. The bacterial destruction of muscle tissue which accelerated the loss of water in cells was slowed down by the blocking function of composite coating, while the hydrophobicity of the citrus essential oil would reduce the water vapor transmission rate in the composite coating and reduce the water evaporation in muscle tissue.

### 3.7 pH

The pH value indicates the acidity and alkalinity of fish meat, reflecting the freshness of fish after death. During different stages after fish death, different biochemical reactions occur in vivo, leading to regular changes of pH in fish.

Figure 3b showed the changes in the pH of the Pacific mackerel during superchilled storage. In the three tested groups, the pH of Pacific mackerel showed a trend of decline after rise. Similar phenomenon has also been obtained for other marine fish species during superchilled storage (Gao et al., 2014). This could be explained by the physicochemical changes in the fish meat after death. The decline of pH in the early stage of superchilled storage may be related to the accumulation of lactic acid, ATP, and phosphocreatine under oxygen‐free environment after the fish died. In the later stage of storage, the value of pH increased again, which is mainly due to the dissolving of endogenous protease in fish body. In the rigor mortis stage after death, there is no significant change in the freshness of fish body.

The results in Figure 3b indicated that the decrease rate of CK in the first day was faster than T1 and T2, and the pH of CK was always lower than T1 and T2. The pH value of these three groups all declined to around 5.75 followed by the rapid increase from the third day. The results of variance analysis showed that from the 6th day, the pH of CK is significantly higher than T1 and T2, while T2 is slightly higher than T1.

The above results showed that chitosan could reduce the decrease of pH on Pacific mackerel in the early stage of superchilled storage, while it could not induce the final reduction of pH. In the later stage of storage, chitosan can alleviate the deterioration of superchilled Pacific mackerel, and reduce the further decomposition of amino acids and other substances. This is possibly due to the isolation effect and chelating agent of chitosan, which can cut off the oxygen and reduce the activity of enzyme. The composition of citrus essential oil occupies the position of some functional groups in the framework of chitosan, enhancing the effect of oxygen isolation, and therefore, the chitosan–citrus coating has a better effect to prevent the Pacific mackerel from corruption.

### 3.8 Thiobarbituric acid‐reactive substances (TBARS)

Pacific mackerel is rich in lipids, which is easy to oxidize and effected by microorganisms. Oxidation of the PUFA would produce substances such as malondialdehyde and ketone. The degree of lipids oxidation can be examined by the value of TBARS.

The results of TBARS value during the superchilled storage are shown in Figure 3c. For all of the three tested groups, TBARS increased fast at first, and the growth rate got lowered in the later period of storage. According to the research of Santiago and Mori (1993), substances like malondialdehyde can cause Maillard reaction with free amino acids in fish, with the storage time extended, the content of free amino acids in Pacific mackerel increases, slowing the accumulation of malondialdehyde. TBARS of CK increased significantly (*p *<* *0.05), while the TBARS in T1 and T2 were less than CK. The contents of TBARS were all <1 mg MDA/Kg in the 0 day, while on the 6th day, the content in CK reached 4.67 ± 0.09 mg MDA/Kg. For T1 and T2, the content of TBARS at the 6th day is only 4.32 ± 0.08 mg MDA/Kg and 4.27 ± 0.08 mg MDA/Kg, respectively. On the 12th day, the content in CK was nearly 1.5 times of the other groups, indicating that the chitosan can prevent the oxidation of unsaturated fatty acids in Pacific mackerel effectively, and it is highly due to the characteristic that chitosan can form film to block oxygen. There were also some differences between T1 and T2. Before the 3rd day, there was no significant difference between the two groups (*p *>* *0.05), and after 3 days, the content of TBARS was significantly higher in T2 than in T1 (*p *<* *0.05), indicating that chitosan matrix added with citrus essential oil can prevent the increase of TBARS content better. In the work of Wu, Fu, et al. (2016), Wu, Li, et al. (2016) and Wu, Wang, et al. (2016), Pacific mackerel was coated with chitosan–gallic acid and stored under 4°C. At the end of storage, the TBARS value of control group and experimental group was 7.93 ± 0.39 mg MDA/Kg and 4.17 ± 0.18 mg MDA/Kg, respectively, similar with the results of this study. Citrus essential oil contains d‐limonene, which has the ability to scavenge free radicals and prevent lipid peroxidation (Singh, Nam, Arseneault, & Ramassamy, 2010). The superposition effect of antioxidant effect in both citrus essential oil and chitosan may be mainly related to the result. In another aspect, the network structure of the composite membrane is more compact than that of chitosan, which reduced the oxygen permeation rate, therefore inhibited the occurrence of the lipid oxidation.

### 3.9 Total volatile basic nitrogen (TVB‐N)

Fish protein tends to be decomposed by endogenous enzymes and microorganisms, and alkalinity substances such as ammonia and amines are produced during this process. Those unstable substances are called total volatile basic nitrogen (TVB‐N), which is an important index of the corruption of fish meat.

The change of TVB‐N value of Pacific mackerel using 3 different methods during superchilled storage was shown in Figure 3d. Generally, the content of TVB‐N had an increasing tendency during superchilled storage. On the first day, the value of TVB‐N in CK and T1 increased slowly. Moreover, the Pacific mackerel was in the period of rigor mortis, the protease in fish released, making the content of TVB‐N increase, but the growth speed of microorganisms was rather slow so that the speed of increase on TVB‐N was slow as well. Later, the TVB‐N values of both the control group and the coated samples increased significantly (*p *<* *0.05) with the extension of storage time. The control samples reached a TVB‐N value of 49.44 ± 1.94 mg/100 g at day 12, and for coating samples, the TVB‐N levels of T1 and T2 groups reached 38.66 ± 4.82 and 30.21 ± 2.67 after 12 days, respectively. It was reported that the TVB‐N limitation of “good quality” was up to 30 mg/100 g (EU/EC, 2008; Jinadasa, 2014). According to this regulation, CK exceeded this limitation at the 6th day, while T1 and T2 reached the limitation at the 12th day and the 15th day, respectively.

These two materials could slow down the increase of TVB‐N in Pacific mackerel as well as the spoilage on Pacific mackerel during superchilled storage. Compared with the two treated groups, the content of TVB‐N in T1 was much higher than T2, indicating that the chitosan adding citrus essential oil had better effect on the superchilled storage of Pacific mackerel and suggested the assistant role of citrus essential oil on antiseptic and fresh keeping. This phenomenon was closely related to the antibacterial property of chitosan–citrus coating, and the enhancement of the barrier performance also reduced the invasion of microorganisms, thereby slowing the rise of TVB‐N in T2.

### 3.10 Total viable count (TVC) and total psychrotrophic count (TPC)

The total number of colonies is an important indicator for fish spoilage. Figure 3e reflected the variation of the total number of colonies in Pacific mackerel during superchilled storage. In all samples, the total viable count (TVC) value of Pacific mackerel was increasing with the extension of storage time. Compared with the control group (CK), the TVC value of experimental groups (T1, T2) was lower, indicating that the reproduction of microorganisms is inhibited in the experiment groups, and this is consistent with the results of other physical and chemical indicators mentioned above. According to the ICMSF (1986), the TVC of 1 × 10^7^ CFU/g is defined as the rejection count. All of the three groups did not reach the limitation at the end of storage; however, the TVB‐N value reached the limitation before TVC, decreasing the quality of Pacific mackerel. Adding citrus essential oil into chitosan enhanced the bacteriostatic effect, and that may be due to the multiplying effect of chitosan and citrus essential oil. d‐limonene, one of the main components of citrus essential oil, is believed to accumulate on the surface of microorganisms, leads to the destruction of membrane integrity and the reduction of proton dynamics and achieve the effect of sterilization. (Sikkema, De Bont, & Poolman, 1994).

The changing regulation on TPC of Pacific mackerel stored under −3°C exhibited similar trends toward TVC, as shown in Figure 3f. It was due to the fact that a majority of the bacterial existed under storage temperature (−3°C) were psychrotrophic bacteria. In the initial storage, the TPC value of the three groups was around 3.6 lg (CFU/g). At the 12th day of storage, the TVC value of CK, T1, and T2 were 5.18 ± 0.032 lg (CFU/g), 4.69 ± 0.084 lg (CFU/g), and 4.46 ± 0.081 lg (CFU/g), respectively. The chitosan–citrus essential oil coating efficiently inhibited the growth of psychrotrophic bacteria during superchilled storage.

### 3.11 Biogenic amines (BA)

Biogenic amines (BA) are ubiquitous in the seafood. However, ingesting large amounts of biogenic amines can lead to physical discomfort. Histamine and tyramine are the main biogenic amines that cause food poisoning. The increase of biogenic amines in food represents the improvement of microbial content. Pacific mackerel belongs to the green peel fish with red meat, which has high content of free amino acids and is prone to catch microorganisms, so the content of biogenic amines is easily exceeded. EC regulated that the histamine content in mackerel should not exceed 100 mg/kg (EC, 2003). Department of Health and Human Services (United States) stipulates that the content of histamine in aquatic products shall not exceed 50 mg/kg (2011). The content of BA is directly related to the degree of corruption.

The changes in biogenic amines content in Pacific mackerel during superchilled storage were shown in Table 3. The contents of all kinds of biogenic amine tested in this study were high in Pacific mackerel, and the content of 5‐serotonin was even up to hundreds of units. Experimental data showed that the content of biogenic amines in Pacific mackerel was rising during the superchilled storage, although there was a slight fluctuation in the data. From the relevant data, the content of biogenic amines in T1 and T2 groups was less than that in CK group, and basically consistent with the law that CK > T1 > T2, for instance, the content of Spd in the 6th day of storage of CK, T1, and T2 was 71.51 ± 2.70 mg/100 g > 21.45 ± 1.80 mg/100 g > 17.34 ± 5.88 mg/100 g, respectively. The data of histamine (His) also showed the same rule. In the storage of Pacific mackerel (6 days), the His content in Pacific mackerel was 190.6 ± 6.21 mg/100 g > 63.30 ± 1.34 mg/100 g > 96.1 ± 6.55 mg/100 g in CK, T1, and T2, respectively. At the 12th day, the histamine content of CK has exceeded the regulation of United States Department of Health and Human Services (2011). Shi, Cui, Lu, Shen, and Luo (2012) and Yu, Xia, Xu, and Jiang (2017) reported that chitosan had the ability to inhibit the growth of microorganisms with histidine decarboxylation activity, and limonene (the major component of citrus essential oil) was also proved to be an inhibitor for histidine decarboxylase (Nitta, Kikuzaki, & Ueno, 2017). The results showed that the chitosan plays an important role in reducing biogenic amine content in Pacific mackerel during superchilled storage, adding citrus essential oil improves the effect.

**Table 3:** Effect of different CS matrix materials of biogenic amines (BA) in frozen Pacific mackerel

BA	Treatment	Storage time (day)					
		0	1	3	6	9	12
Put	CK	14.68 ± 3.34	22.22 ± 1.29	14.36 ± 2.08	25.13 ± 1.37	66.72 ± 1.82	62.06 ± 2.58
	T1	15.65 ± 1.47	34.68 ± 0.005	14.94 ± 2.87	39.03 ± 1.15	47.17 ± 1.57	67.08 ± 1.35
	T2	16.29 ± 3.50	19.20 ± 3.07	12.23 ± 1.65	26.95 ± 1.82	50.33 ± 1.03	56.57 ± 1.31
Cad	CK	5.09 ± 1.63	11.07 ± 0.28	6.57 ± 1.71	15.16 ± 2.11	31.39 ± 0.82	37.37 ± 1.20
	T1	5.16 ± 0.82	10.75 ± 1.11	6.27 ± 1.01	13.64 ± 0.73	18.63 ± 1.84	26.51 ± 0.37
	T2	8.22 ± 0.32	6.56 ± 2.06	6.47 ± 0.09	10.85 ± 2.03	26.14 ± 0.88	30.73 ± 0.84
Spd	CK	15.36 ± 1.63	32.2 ± 1.15	71.51 ± 2.70	88.86 ± 0.98	86.63 ± 0.76	154.6 ± 2.67
	T1	15.93 ± 2.17	32.32 ± 0.83	21.45 ± 1.80	83.14 ± 1.98	98.39 ± 1.03	82.02 ± 2.00
	T2	14.21 ± 1.13	37.72 ± 7.61	17.34 ± 5.88	33.42 ± 1.88	24.03 ± 0.38	23.97 ± 0.14
Spm	CK	83.42 ± 5.65	92.91 ± 3.11	89.35 ± 2.13	101.26 ± 1.98	125.24 ± 1.56	86.19 ± 2.26
	T1	78.78 ± 4.69	150.02 ± 4.09	79.33 ± 0.18	90.71 ± 0.59	102.61 ± 3.19	172.4 ± 2.15
	T2	60.64 ± 0.15	88.03 ± 8.96	77.54 ± 2.56	72.00 ± 1.33	134.78 ± 1.32	53.41 ± 1.83
Try	CK	75.76 ± 1.54	37.55 ± 1.99	47.30 ± 1.01	133.97 ± 3.56	223.19 ± 4.23	234.7 ± 1.86
	T1	31.50 ± 1.70	28.31 ± 1.78	44.05 ± 1.09	114.43 ± 2.11	135.39 ± 3.11	224.3 ± 4.95
	T2	30.84 ± 5.70	22.62 ± 0.79	30.12 ± 1.66	60.74 ± 1.76	160.02 ± 3.81	110.8 ± 2.08
Ser	CK	62.05 ± 9.96	67.78 ± 3.85	63.36 ± 3.38	161.61 ± 2.39	199.23 ± 1.92	50.43 ± 0.06
	T1	80.11 ± 0.01	60.91 ± 3.50	71.54 ± 0.25	51.24 ± 1.98	51.17 ± 0.84	52.60 ± 1.96
	T2	76.70 ± 4.05	58.15 ± 2.95	68.06 ± 1.95	54.98 ± 1.52	74.49 ± 0.24	51.13 ± 1.04
Tyr	CK	155.5 ± 7.75	137.4 ± 4.64	79.13 ± 2.62	68.98 ± 0.97	76.53 ± 0.34	125.6 ± 0.78
	T1	195.6 ± 6.97	78.51 ± 3.55	66.60 ± 1.74	63.04 ± 1.45	65.19 ± 1.54	69.55 ± 3.98
	T2	100.6 ± 7.19	115.3 ± 1.34	83.07 ± 2.87	64.36 ± 0.12	70.45 ± 1.36	63.12 ± 2.77
His	CK	14.47 ± 2.18	101.3 ± 3.00	123.6 ± 2.62	190.6 ± 6.21	361.6 ± 1.36	515.2 ± 1.95
	T1	54.07 ± 1.15	63.30 ± 1.34	93.54 ± 2.65	108.5 ± 2.73	262.2 ± 4.36	395.3 ± 2.31
	T2	17.17 ± 0.95	20.47 ± 2.34	82.14 ± 1.23	96.1 ± 6.55	193.3 ± 3.66	303.4 ± 4.32

### 3.12 Correlation coefficients between different indicators of Pacific mackerel during superchilled storage

The correlation coefficient between different indicators (TBARS, TVC, TVB‐N, pH, and DL) of Pacific mackerel during superchilled storage was analyzed, and the results were shown in Table 4. In all of 3 tested groups (CK, T1, T2), most of the indicators showed a consistent performance trend that the value increases with the extend of storage time. The correlation coefficients among TBARS, TVC, TVB‐N, and DL are all above 0.776. These four indicators showed the same trend during the process of fish spoilage. However, the value of pH does not perform evident correlation between other indicators. That might due to the decrease of pH in the early stage of superchilled storage caused by the accumulation of lactic acid. As a result, pH is not a convincing indicator for evaluating chemical changes of Pacific mackerel. This statement is agreed with Li et al. (2011), Wu, Fu, et al. (2016), Wu, Li, et al. (2016) and Wu, Wang, et al. (2016).

**Table 4:** Correlation coefficients between different indicators of Pacific mackerel during chilled storage

Samples	Indicator	TBARS	TVC	TVB‐N	pH	DL
CK	TBARS	1.000				
	TVC	0.943^a^	1.000			
	TVB‐N	0.945^a^	0.966^a^	1.000		
	pH	0.459	0.550	0.685	1.000	
	DL	0.987^a^	0.898^b^	0.891^b^	0.325	1.000
T1	TBARS	1.000				
	TVC	0.813^b^	1.000			
	TVB‐N	0.892^b^	0.952^a^	1.000		
	pH	0.289	0.784	0.616	1.000	
	DL	0.985^a^	0.865^b^	0.938^a^	0.459	1.000
T2	TBARS	1.000				
	TVC	0.913^a^	1.000			
	TVB‐N	0.802^b^	0.888^a^	1.000		
	pH	0.091	0.718	0.549	1.000	
	DL	0.977^a^	0.776^b^	0.905^a^	0.197	1.000

*Note*

^a^Significance at 5% level. ^b^Significance at 1% level.

### 3.13 Sensory evaluation

**Figure 4:**
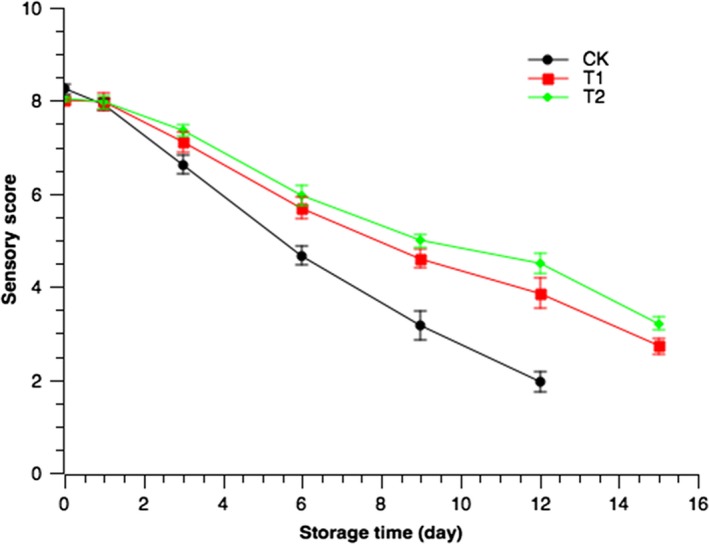
Sensory evaluation

The sensory quality of Pacific mackerel was shown in Figure 4. During the whole storage period, the sensory scores of all samples showed a downward trend. In the 0 day, the sensory scores of experimental groups were lower than that of CK. This may be due to the acidity of the coating solution. Then, during the storage, the sensory scores of experimental groups were higher. The sensory scores of Pacific mackerel coated with chitosan–citrus essential oil were higher than those of other groups, which may be due to the stronger antioxidant and antimicrobial properties of chitosan–citrus essential oil. Although some citrus essential oil odor could be noticed in T2, in general, the sensory acceptability of the samples over the entire storage period followed the order T2 > T1 > CK, which was consistent with the results of Rao et al. (2017). The coating of chitosan also weakened the release of odor (Sao Pedro, Cabral‐Albuquerque, Ferreira, & Sarmento, 2009).

## 4 CONCLUSION

The antioxidant and antibacterial ability of chitosan–citrus essential oil composite coating and its ability to preserve the quality of Pacific mackerel during superchilled storage are discussed in this study. Chitosan–citrus has good superoxide anion radical scavenging activity as well as hydroxyl radical scavenging activity with fine antibacterial property. Chitosan–citrus composite coating has better inhibition effect on microbial growth, and it can alleviate lipid oxidation and peroxide production. Adding of citrus essential oil has a more significant effect on controlling the deterioration of the quality of Pacific mackerel during superchilled storage than using chitosan alone. This was supposed to be related to the bacteriostatic effect of citrus essential oil as well as the superposition effect of these two materials. In this study, the chitosan–citrus essential oil composite coating could extend the shelf life of Pacific mackerel for around 3 days. The combination of chitosan with citrus essential oil may be a promising way for maintaining the storage quality of Pacific mackerel. Chitosan–citrus coating has the great potential for the usage in food industry as a food‐grade bio‐preservative.

## CONFLICT OF INTEREST

The authors declare that they do not have any conflict of interest.

## AUTHOR CONTRIBUTIONS

Yuan Li: Write paper and do most of the experiments. Chunhua Wu: Design the experiments. Tiantian Wu: Do the data analyzing part. Chunhong Yuan: Language polishing and give useful advice on the experiments. Yaqin Hu: Give overall guidance on the experiments and support funding.

## ETHICAL REVIEW

This study does not involve any human or animal testing.

## INFORMED CONSENT

Written informed consent was obtained from all study participants.
